# Rearranged T Cell Receptor Sequences in the Germline Genome of Channel Catfish Are Preferentially Expressed in Response to Infection

**DOI:** 10.3389/fimmu.2018.02117

**Published:** 2018-09-27

**Authors:** Robert Craig Findly, Frank D. Niagro, Ryan P. Sweeney, Alvin C. Camus, Harry W. Dickerson

**Affiliations:** ^1^Department of Infectious Diseases, College of Veterinary Medicine, University of Georgia, Athens, GA, United States; ^2^Department of Pathology, College of Veterinary Medicine, University of Georgia, Athens, GA, United States

**Keywords:** T cell receptor, rearranged VDJ genes, germline genome, DNA transposition, channel catfish

## Abstract

Rearranged V(D)J genes coding for T cell receptor α and β chains are integrated into the germline genome of channel catfish. Previous analysis of expressed TCR Vβ2 repertoires demonstrated that channel catfish express multiple public clonotypes, which were shared among all the fish, following infection with a common protozoan parasite. In each case a single DNA sequence was predominately used to code for a public clonotype. We show here that the rearranged VDJ genes coding for these expressed public Vβ2 clonotypes can be amplified by PCR from germline DNA isolated from oocytes and erythrocytes. Sequencing of the Vβ2 PCR products confirmed that these expressed public Vβ2 clonotypes are integrated into the germline. Moreover, sequencing of PCR products confirmed that all five Vβ gene families and Vα1 have rearranged V(D)J genes with diverse CDR3 sequences integrated into the germline. Germline rearranged Vβ2 and Vβ4 genes retain the intron between the leader and Vβ sequence. This suggests that the germline rearranged TCR Vβ genes arose through VDJ rearrangement in T cells, and subsequently moved into the germline through DNA transposon mediated transposition. These results reveal a new dimension to the adaptive immune system of vertebrates, namely: the expression of evolutionarily conserved, rearranged V(D)J genes from the germline.

## Introduction

The vertebrate adaptive immune system has evolved to counter an unpredictable and constantly evolving array of antigens presented by pathogens. The theoretical diversity of the antigen binding domain of the murine αβ T cell receptor (TCR) is predicted to encompass ~10^15^ different aa combinations and that of the human β chain is estimated at ~5 × 10^11^ aa sequences (1, 2). This diversity is generated through somatic rearrangements that result in the joining of a Vα gene and Jα gene from the families of Vα and Jα genes and, likewise, a Vβ gene with a Dβ and Jβ gene from the multiple Vβ and Jβ genes. It is augmented by non-templated deletion and addition of nucleotides at the Vα-Jα junctions, or Vβ-Dβ and Dβ-Jβ junctions. These junctional sequences code for the region of greatest sequence diversity in the antigen binding domain of the TCR, which is defined as the third complementarity determining region (CDR3). The CDR3 of the B cell receptor (BCR) is formed similarly (1). These unique CDR3 amino acid sequences specify the clonotypic landscape of vertebrate T and B cell populations.

The extent to which this potential repertoire diversity is expressed has been accessed by high throughput sequencing of TCRβ CDR3 sequences, either from rearranged TCRβ genes in genomic DNA isolated from αβ T cells, or from cDNA libraries prepared from expressed transcripts. This showed that although only a limited fraction of the potential diversity is expressed at any one time, the repertoire is not static. Limited sharing of CDR3 nucleotide sequences was detected among individuals, but greater than statistically predicted sharing was observed for TCRβ CDR3 aa sequences in humans and mice and of expressed IgH CDR3 sequences in zebrafish. This is attributed to convergent recombination (2–7). It also demonstrated that there is differential utilization of specific Vβ and Jβ genes. The possibility has been previously considered that highly conserved, public TCRβ CDR3 aa sequences, coded by sequences with few non-templated junctional sequences, are expressed from rearrangement of specific Vβ, Dβ, and Jβ gene sequences that have been selected for, as they recognize common epitopes from pathogens, such as Epstein-Barr virus (2, 3).

**Figure 1:**
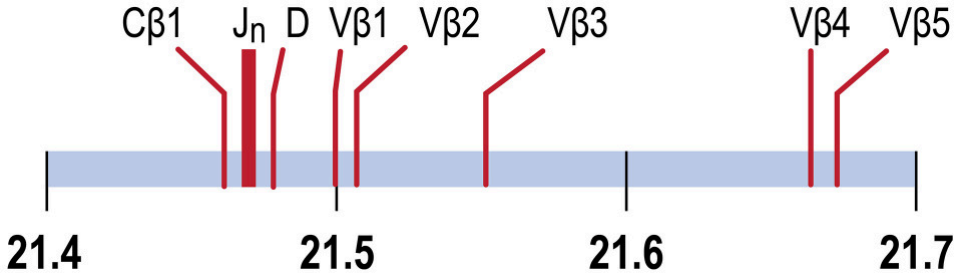
TCRβ genes map to chromosome 9. The positions of the Cβ1 gene, the 29 Jβ genes, the Dβ gene and Vβ genes 1, 2, 3, 4 and 5 are indicated. Vβ5 is separated from Cβ1 by 204.4 Kb. Units in Mb.

In the channel catfish (*Ictalurus punctatus*), a teleost model of early vertebrate adaptive immunity, a partial sequence of the TCRβ locus identified a single Dβ gene, 29 Jβ genes and two Cβ genes, *TCRC*β*1* and *TCRC*β*2*. *TCRCB1* is predominantly expressed. cDNA sequences for twelve Vβ genes, organized in five families, have been determined (8, 9). Although the channel catfish genome has been sequenced, the TCRβ genes have not been annotated (10, 11). We have mapped the TCRβ genes to chromosome 9, spanning a region of ~204 kb, arranged in a translocon organization (Figure 1). Introns separate leader sequences from Vβ genes for all five Vβ families. The TCRα genes map to chromosome 7. The Vβ and Jβ gene families coding for channel catfish TCRβ are arranged in a translocon organization similar to other teleosts including puffer fish, (*Tetraodon nigroviridis*), Atlantic salmon (*Salmo salar)*, and zebrafish (*Danio rerio*) (8, 9, 12–14).

We have previously used high-throughput sequencing to examine the expressed diversity of TCR Vβ2 CDR3 repertoires in different tissues of outbred channel catfish before and after an immunizing infection with the protozoan parasite *Ichthyophthirius multifiliis*. Because antibodies recognizing channel catfish αβ T cells have not been developed, we could not directly isolate αβ T cells from tissues. Instead, we isolated RNA from tissue samples and sequenced expressed TCRβ CDR3 sequences in cDNA libraries. We define public clonotypes as those shared among all the fish (15). We identified public clonotypes whose expression increased after infection with the most abundant of these accounting for up to ~8% of all copies of CDR3 sequences in a tissue. Because of codon degeneracy we expected that multiple DNA sequences would code for the CDR3 of each of these abundant public clonotypes (2–7). Instead, we found that a single DNA sequence was predominately used to code for the CDR3 of each public clonotype in individual fishes. Moreover, this identical DNA sequence was predominately used to code for the CDR3 of that same clonotype in all four fish sampled. This suggested that these CDR3 sequences were not generated through standard VDJ rearrangements, but that transcriptionally active rearranged TCRβ VDJ gene sequences were stably integrated into the *I. punctatus* germline genome (15).

To determine if sequences for rearranged TCR Vβ2 genes coding for these public clonotypes could be amplified by PCR from germline DNA of channel catfish we designed primers for the Vβ2 gene and public CDR3 sequences. Germline DNA was isolated from oocytes and erythrocytes, which are nucleated in fish. PCR products of the predicted sizes were amplified from germline DNA and sequencing confirmed that rearranged VDJ genes coding for these public Vβ2 clonotypes are integrated into the germline genome. We extended this to show that sequences for rearranged Vβ-Dβ-Jβ genes coding for all five Vβ gene families and a Vα1–Jα gene are integrated into the germline genome. The germline sequences of all five Vβ gene families have an intron between the leader and the Vβ gene and sequencing confirmed that this intron sequence is retained in the rearranged Vβ2 and Vβ4 VDJ sequences in the germline.

Rearranged genes coding for αβ TCRs, integrated into the germline genome, have not been previously described in teleosts. A rearranged TCR, designated TCRμ, is present in the germline of a marsupial, arranged in a cluster configuration with each cluster containing a Vμ, Dμ, Jμ, fused VμDμJμ (Vμ_j_) and Cμ gene segments. The leader sequence preceding the fused Vμ_j_ gene is not separated from the Vμ by an intron, leading to the proposal that the fused Vμ_j_ gene originated by RAG driven VDJ recombination in a T cell followed by retrotransposition into the germline (16). A rearranged IgH VDJ gene with a single open reading frame is present in the germline genome of channel catfish adjacent to a Tc1/mariner transposable element, although it is not known if it is transcribed (17, 18).

In bony fish IgL genes are arranged in clusters, but the V and J are not found in a fused configuration (19). In cartilaginous fish the genes for IgH and IgL chains are arranged in a cluster configuration. In some species of sharks the IgL V and J genes, or IgH V, D and J genes, within a cluster are organized in the germline as separate gene segments, or in fused VJ, VD-J or VDJ configurations with the fused genes exhibiting sequence diversity at their junctions. The origin of the fused genes in the germline of cartilaginous fish is attributed to RAG activity in germ cells (20, 21). The fused genes in sharks are only expressed early in development and were postulated to protect against infection by commonly encountered pathogens (21).

In contrast to sharks, we hypothesize that in channel catfish the germline rearranged TCR genes were generated by somatic recombination in developing αβ T cells and then subsequent transposition into the germline genome. As the rearranged genes contain the intron sequences between the leader and Vβ sequences, this suggests that transposition was mediated by a DNA transposon and not through retrotransposition (22). These rearranged TCR genes include those that code for public Vβ2 clonotypes that are preferentially expressed in mature fish after infection with the protozoan parasite *I. multifiliis* (15). We propose that these germline rearranged TCRs are expressed by a novel class of αβ T cells, enabling rapid mobilization of populations of αβ T cells that express clonotypes which recognize and bind antigens expressed during infections with commonly encountered pathogens, such as *I. multifiliis*. The αβ T cells expressing these germline clonotypes function as a novel, evolutionarily conserved class of T cell, which we designate as a “germline T cell.” This represents a new paradigm for vertebrate adaptive immune responses in which αβ T cells expressing rearranged TCR genes, which are integrated into the germline genome, are preferentially expanded during a primary infection.

## Materials and methods

### Channel catfish

Outbred channel catfish (*Ictalurus punctatus*) fingerlings, ~4 months of age and weighing ~5 g, were obtained from both state and private fish hatcheries in Georgia, and raised as previously described (23, 24). All animal experiments were approved by the University of Georgia IACUC.

### Isolation of oocytes

Female channel catfish (120 to 350 g) were euthanized in 0.07% Tricaine, 0.07% NaHCO_3_. Ovaries were removed by dissection, washed four times in 50 mM KH_2_PO_4_, 150 mM NaCl, pH 7.2, the ovarian membrane removed, and oocytes teased apart. Oocytes were incubated overnight with 0.1 % type A collagenase (Sigma) in 50 mM KH_2_PO_4_, 150 mM NaCl, pH 7.2 at RT with gentle shaking, and washed five times in the same buffer without collagenase. From 90 to 190 oocytes from a fish were collected by hand, incubated overnight at 56°C in proteinase K, and ATL lysis buffer (Qiagen), and total oocyte DNA isolated using DNeasy mini spin columns per manufacturer's instruction (Qiagen DNeasy).

### Isolation of erythrocytes by laser dissection microscopy

Blood was collected from individual fish by venipuncture of the caudal vein using a 21 gauge needle and tuberculin syringe. The needle was removed and blood gently expressed into a 400 μl heparinized Microtainer tube (Becton-Dickinson). Blood smears were prepared by spreading a drop of blood onto the surface of an RNAse free MMI membrane slide and support (Molecular Machines & Industries). Smears were allowed to air dry and stained using a Hema-3 stain kit (Fisherbrand). To prevent cross-contamination, smears were stained by individually flooding slide surfaces, each with fresh reagents, rather than by dipping them into a common staining jar. Slides were examined at 400 X using an Olympus IX71 microscope and erythrocytes excised using an MMI CellCut Plus laser microdissection and isolation cap system (Molecular Machines & Industries). Only widely separated erythrocytes were excised near the feather edge of the smear. Approximately 100–150 erythrocytes were collected per isolation tube. DNA was isolated from erythrocytes using DNeasy mini spin columns per manufactures instruction (Qiagen DNeasy).

### Primer sequences

Sequences for twelve Vβ genes, Dβ, Jβ, Cβ genes, and Vα and Jα genes of channel catfish have been determined (8, 9, 25). The primer sequences used for PCR amplification of Vβ and Vα genes are listed in Table 1 and Jα and Jβ genes in Table 2. Primers for CDR3 sequences coding for Vβ2 public clonotypes are listed in Table 3. The primers sequences for Vβ leader and CDR3 sequences are listed in Table 4.

**Table 1:** List of V gene primer sequences.

**V Genes**	
**Gene**	**F Primer 5′to 3′**
Vβ1	GGTATCGGCAAAACAGTCGT
Vβ2	TCCAGTGCAGTCACAATGACAA
Vβ2	GTAACACAGTTATGGCACTGATTG
Vβ3	ACTGAGCCAAAGAACGAGGA
Vβ4	GACTTTGGAAAATCCGACCA
Vβ5	CAGCCAGGACACAGGATTTA
Vα1 TS.32.34	AACAACCAGTCCTACAGGAAACT

**Table 2:** List of J gene primer sequences.

**J Genes**	
**Gene**	**R Primer 5′to 3′**
Jβ3	TGAGTTTTGTTCCAGCTCCA
Jβ12	CTCTTACAGTCAGTTTAGTTCCA
Jβ15	GAACTGTTAACTTGGTTCCTCCA
Jβ18	AGAACTGTTAACTTGGTTCCTCC
Jβ21	CAAGAACGGTTAGTTTGGT
Jβ24	CAAGAACTGTGAGTTTGGTCCC
Jβ27	ACTGTTAACTTGGTCCCTTCAC
Jβ28	CTATCACTGTGAGTTTGGTGCCA
Jα TS.32.34	GCTTCCAGTAGTAGGCACCAGC

**Table 3:** List of CDR3 primers for Vβ2 public clonotypes.

**Clonotype and 5′to 3′CDR3 sequence**	**Primer 5′to 3′**
CAAHRGANPAYF TGTGCAGCCCACAGGGGGGCCAATCCAGCATACTTT	R: CCTGTGGGCTGCACAGTA
CAAIMGGTQPAYF TGTGCAGCCATAATGGGTGGCACTCAGCCTGCATACTTT	F: ATAATGGGTGGCACTCAGC R: AGGCTGAGTGCCACCCATTA
CAAKDRGLSSPAYF TGTGCAGCCAAAGACAGGGGTCTCAGTTCGCCAGCATATTTT	R: CCTGTCTTTGGCTGCACA
CAAKQISGVNPAYF TGTGCAGCCAAGCAGATCTCTGGAGTCAATCCAGCTTACTTC	F: GCAGATCTCTGGAGTCAATC R: TTGACTCCAGAGATCTGCTT
CAARKAYGNNPAYF TGTGCAGCCAGAAAAGCTTATGGAAACAATCCAGCTTACTTT	F: GAAAAGCTTATGGAAACAATC R: AAGCTTTTCTGGCTGCACA
CAARKDKYEAYF TGTGCAGCCAGAAAAGACAAATATGAGGCCTATTTC	R: TGTCTTTTCTGGCTGCACA
CAARQLTNTYPAYF TGTGCAGCCAGACAGCTAACAAACACCTATCCTGCTTACTTT	F: GACAGCTAACAAACACCT R: TAGCTGTCTGGCTGCACA
CAARTTGSNPAYF TGTGCAGCCAGAACCACAGGGAGCAACCCAGCATACTTT	F: AGAACCACAGGGAGCAACC

**Table 4:** List of primer sequences for Vβ leader and CDR3 sequences.

**Gene**	**Leader primer 5′to 3′**	**CDR3 primer 5′to 3′**
Vβ1	TGTGGACTCTGTGTTGTCTTCA	AGCTTGACTGGCCTGAGTCC
Vβ2	GTGAACTCATCGTGTTCTTCA	Table 3
Vβ3	CTAAGTTGTGCCTGTTCTTG	AGCTTGATTGGCACCAATCC
Vβ4	CAGTCACTTTACTGGATTCAAGGAG	CAGAGAATTCTCTCACGGCACA
Vβ5	GCCACACTATTATGTCTTGCAG	CAGAGCCGGCTACACAGAA

### PCR and DNA sequencing

A 5 μl sample of oocyte DNA preparations corresponded to ~2 to 5 oocytes, and 5 μl of erythrocyte DNA preparations to ~2 to 4 erythrocytes. PCR was carried out in 20 μl using 5 μl of oocyte or erythrocyte DNA, 0.25 μM forward and reverse primers, 0.75 mM MgSO4, and 12.5 μl of Platinum Hot Start PCR Master Mix (Invitrogen). Reactions were incubated at 94°C for 2 min, 30 cycles of 94°C for 30 s, 61°C for 30 s, and 68°C for 30 s, with a final extension at 68°C for 5 min in a T100 thermal cycler (BioRad). This PCR protocol was repeated using 5 μl of cDNA from the first round of PCR for 30 cycles, followed by an additional 15 cycles using 5 μl of cDNA from the second round of PCR. Selected PCR products were recovered from 2 % agarose gels using ZymoClean Gel DNA Recovery Kit. PCR products were purified with SPRI beads and sequenced by the University of Georgia Genomics Facility, or Genewiz, using Big Dye Terminator 3.1 kit with an Applied Biosystems 3730xl sequencer. NCBI BLAST was used to determine sequence similarities.

### Chromosome sequences

UGene 1.26 was used to assign TCR sequences to chromosomes 7 and 9.

## Results

### Rearranged Vβ to Jβ sequences coding for public Vβ2 clonotypes are integrated into the germline genome

Germline DNA was isolated from channel catfish oocytes, including both germinal vesicle and mitochondrial DNA, to determine if sequences coding for rearranged TCRβ genes of public clonotypes could be amplified by PCR from the germline genome. A similar PCR approach has been used to measure TCRβ CDR3 sequence diversity in genomic DNA isolated from human CD8^+^ T cells (2). The dominant CDR3 sequences for six of the previously identified public clonotypes were used to design primers specific to each clonotype (15). Reverse PCR primers complementary to the CDR3 sequences coding for these six public clonotypes were individually paired with a forward Vβ2 gene primer, which resulted in amplification of PCR products of the predicted length for all six clonotypes from oocyte DNA. Sequencing of the PCR products demonstrated that each of these genomic CDR3 sequences was identical to its respective transcribed CDR3 sequence for all six clonotypes (Table 5, Supplemental Table 1).

**Table 5:** Vβ2 public clonotypes integrated into the germline genome.

	**Oocyte sequence #**					**Erythrocyte sequence #**		**PCR CDR3-J**β	
**Vβ2 Clonotype**	**Jβ Gene**	Fish 1^*^	Fish 9^*^	Fish H1^*^	Fish 6^*^	Fish H1^*^	Fish D1^*^	Fish 1^*^	Fish 6^*^
CAAHRGANPAYF	3	+	+	+					
CAAIMGGTQPAYF	24	+	+	+	+	+	+	+	+
CAAKDRGLSSPAYF	25	+	+	+					
CAARKAYGNNPAYF	21	+^*^	+	+	+				+
CAARKDKYEAYF	7	+	+	+					
CAARQLTNTYPAYF	12	+	+	+					+
CAAKQISGVNPAYF	18							+	+
CAARTTGSNPAYF	28							+	+

+ *Designates those fish that were sampled by PCR and PCR products sequenced to confirm that the clonotype was present*.

# *Sequence data shown in Supplementary Information*.

* *Individual fish identification number*.

**Figure 2:**
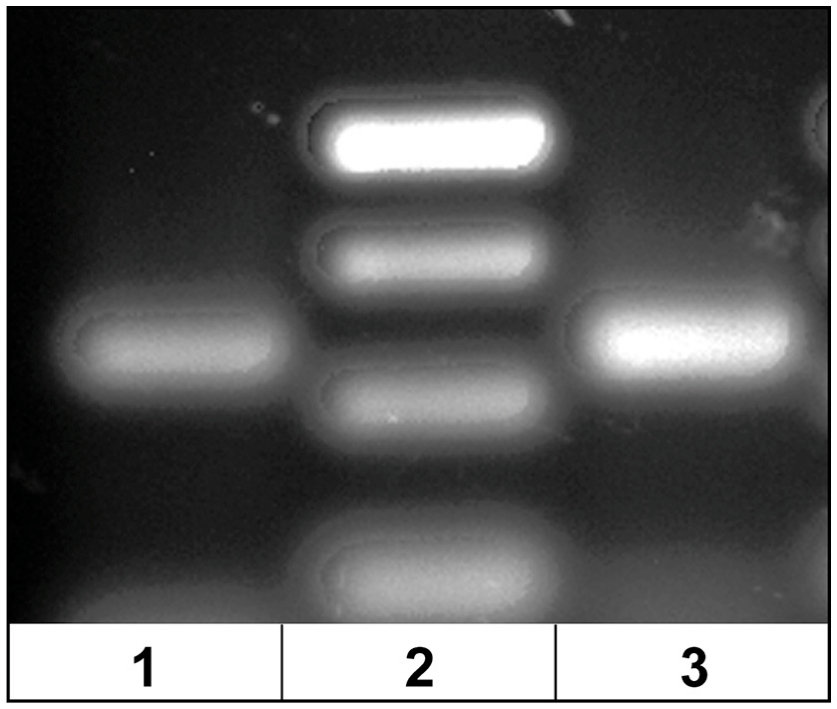
PCR products amplified from oocyte DNA using primer pairs for the CDR3 sequence of a public clonotype and its Jβ partner. (1) Clonotype CAAKQISGVNPAYF with J18 (58 bp), (2) GeneRuler low range ladder (Thermo-Fisher) and (3) clonotype CAAIMGGTQPAYF with J24 (59 bp).

The dominant Vβ2–CDR3 sequence for each of these public clonotypes was previously shown to be transcribed in combination with the same J gene (15). If these same CDR3–Jβ gene combinations were present in germline DNA, then using primers complementary to a CDR3 sequence coding for a public clonotype and its Jβ gene partner should amplify short ~50-60 bp products from oocyte DNA. For the five public clonotypes tested, the combination of a CDR3 primer with its Jβ partner amplified an ~50–60 bp PCR product, as shown for clonotypes CAAIMGGTQPAYF and Jβ24, or CAAKQISGVNPAYF and Jβ18 (Figure 2). The PCR products were restricted to the Jβ gene associated with each public clonotype, as no PCR products were observed when the CAAIMGGTQPAYF primer was paired with primers for Jβ12 or Jβ21. Similarly, the primer for clonotype CAARKAYGNNPAYF paired with Jβ21 amplified a 60 bp band, but no band was observed when it was combined with primers for Jβ12 or Jβ24. Likewise, for clonotype CAARQLTNTYPAYF a product was observed in combination with Jβ12, but not with Jβ21 or Jβ24. These results suggested that rearranged Vβ-Jβ sequences for these public clonotypes are integrated into germline DNA (Table 5).

### All five Vβ gene families have rearranged Vβ to Jβ sequence integrated into the oocyte germline genome

To explore the possibility that rearranged VDJ genes for all Vβ gene families are integrated into the germline genome, primers specific for each of the five Vβ gene families (Vβ1 to Vβ5) were tested in multiplex combinations with Jβ gene primers to determine if PCR products of 200 to 300 bp could be amplified from oocyte DNA. The 29 Jβ genes fall into seven groups with the members of a group sharing similar or identical sequences 3′ of the conserved phenylalanine codon. Consequently, a Jβ gene primer complementary to a 3′ Jβ sequence can anneal to other Jβ genes in its group, in contrast to the gene specific Vβ primers. We were concerned that PCR amplification of Vβ-Jβ combinations containing identical Vβ and Jβ sequences, but different CDR3 sequences, would preclude direct sequencing of full length PCR products. Indeed, PCR products for some Vβ-Jβ combinations did contain multiple sequences and accurate sequences could not be determined. Nevertheless, we obtained double-stranded sequences and one single-stranded sequence of PCR products for eleven Vβ-Jβ combinations. These rearranged VDJ gene sequences included all five Vβ gene families (Table 6, Supplemental Table 2).

**Table 6:** Clonotypes of integrated TCR V to J genes in genomic DNA isolated from oocytes: five Vβ gene families and Vα1.

**V Gene sequence #**	**CDR3 clonotype**	**Primers**	**Fish ID number‡**
Vβ1-Jβ13	CAASPGGASQAYF	Vβ1-Jβ18	8
Vβ1-Jβ13^*^	CAASQSGRTQASQAYF	Vβ1-Jβ18	9
TB4-Jβ21 Vβ1 family	CAASIDGNNPAYF	Vβ1-Jβ21	3
Vβ2-Jβ27	CAAKITGDYSLQAYF	Vβ2- Jβ15	8
Vβ2-Jβ13	CAARTGISGGASQAY	Vβ2- Jβ18	8
Vβ3-Jβ17	CAARDGQGIGANQAYF	Vβ3-Jβ15	H6
Vβ3-Jβ17^*^^+^	CAAQGGGANQAYF	Vβ3-Jβ27	9
Vβ4-Jβ20	CAVREYNGGREAYF	Vβ4- Jβ18	3
Vβ4-Jβ20^*^	CAVREFSGGREAYF	Vβ4-Jβ18	H7
Vβ4-Jβ11	CAGEIV??QA??	Vβ4-Jβ18	9
Vβ5-Jβ2/3	CVAFPGQGFTGSAYF	Vβ5-Jβ3	8
Vβ5 like-Jβ17	CAANSGGANQAYF	Vβ5-Jβ27	8
Vα1	CALV??TG	VαTS.32.34 Jα TS.32.34	8
Vα1	CALV	VαTS.32.34 Jα TS.32.34	H1
Vα1	CALV	VαTS.32.34 Jα TS.32.34	H6

# *Sequence data shown in Supplementary Information*.

* *Same CDR3 sequence in erythrocyte*.

+ *Forward primer sequence only*.

‡ *Individual fish identification number*.

A combination of Vβ1–Jβ18 primers amplified PCR products with different CDR3 sequences from oocyte DNAs of two fish. The Vβ sequences were 99% similar to Vβ1, but matched Jβ13, which is in the same Jβ group as Jβ18. Oocyte DNA from a third fish was used as a template for Vβ1–Jβ21 primers and the 240 bp product had 85% similarity to Vβ1, but 96% similarity to TB4, a another Vβ1 family gene. Vβ2–Jβ15 primers generated a 290 bp Vβ2–Jβ27 sequence with 99% similarity to Vβ2 and a CDR3 aa sequence of CAAKITGDYSLQAYF. This same oocyte DNA amplified with Vβ2–Jβ18 primers generated a 277 bp Vβ2–Jβ13 sequence with 99% similarity to Vβ2 and a CDR3 aa sequence of CAARTGISGGASQAY. The sequences coding for these clonotypes were not detected in our high-throughput cDNA sequence libraries, which indicates that either not all integrated sequences were transcribed after *I. multifiliis* infection, or that our Illumina data set was not sufficiently large. A Vβ3–Jβ15 primer pair generated a Vβ3–Jβ17 product that showed 98% similarity to Vβ3. Only single-stranded sequence was obtained from a Vβ3–Jβ27 primer pair that amplified a Vβ3–Jβ17 product, which showed 98% similarity to Vβ3. For Vβ4, three PCR products with different CDR3 sequences were amplified using Vβ4–Jβ18 primers and oocyte DNA collected from three fish. They had 97% to 99% similarity to Vβ4. A Vβ5–Jβ3 primer pair generated a sequence with 100% similarity to Vβ5. In contrast, Vβ5–Jβ27 primers used with the same oocyte DNA amplified a sequence for clonotype CAANSGGANQAYF and had with only 79% similarity to Vβ5 (Table 6).

Only some Vβ-Jβ primer combinations resulted in amplification of a PCR product, which indicates that not all possible combinations of rearranged Vβ-Jβ genes are integrated into the germline genome. The rearranged TCR Vβ-Jβ sequences incorporated CDR3 sequences with random sequence diversity at both Vβ-Dβ and Dβ-Jβ junctions when compared to their respective Vβ and Jβ genomic sequences. This suggests that these integrated Vβ to Jβ sequences originated during somatic rearrangements of a Dβ gene to a Jβ gene followed by a Vβ gene to the fused Dβ-Jβ sequence in the course of αβ T cell development (26).

### All five Vβ gene families have rearranged Vβ to Jβ sequence integrated into the erythrocyte germline genome

**Figure 3:**
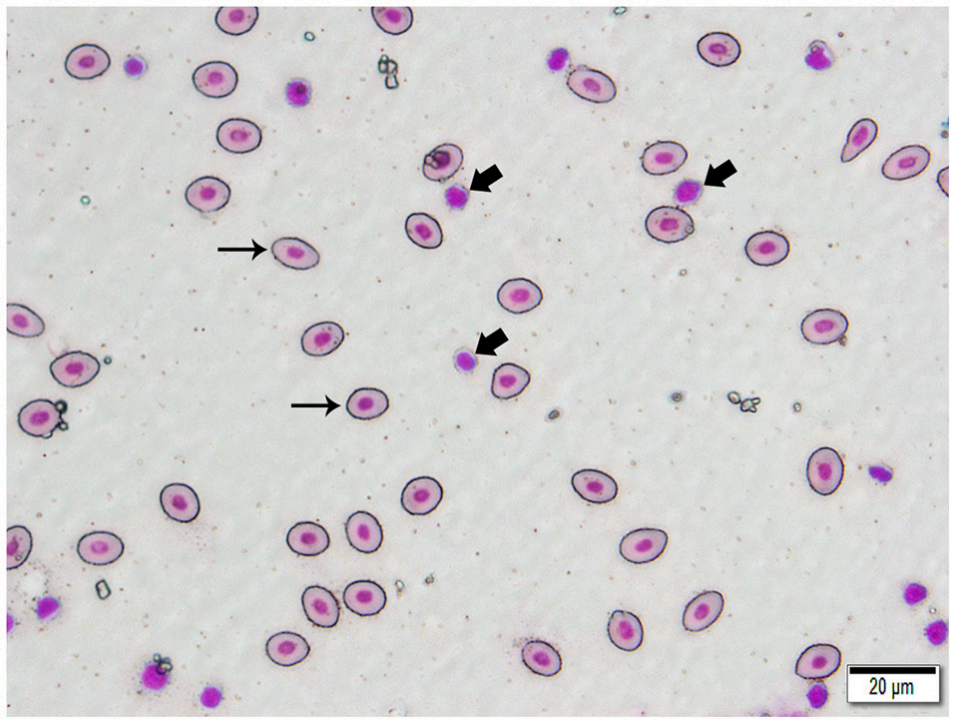
Photomicrograph of a typical 400 X field of channel catfish blood prepared for laser capture microdissection and stained with a Wright-Giemsa type stain. Individualized, large, oval erythrocytes (thin arrows) were excised by MMI CellCut Plus laser microdissection. Lymphocytes (thick arrows), in proximity to erythrocytes were readily visualized and not included in the cell collection. (Hema-3 stain, bar = 20 μm).

We isolated DNA from erythrocytes to confirm our results obtained using oocyte DNA. Erythrocytes are nucleated in channel catfish, as in all teleosts, and represent a second cell type in which the genes coding for the TCR should not have undergone somatic rearrangement. To insure that erythrocyte preparations were not contaminated with T cells, individual erythrocytes were isolated from blood smears by laser dissection microscopy (Figure 3). DNA was isolated from pools of 100 to 150 erythrocytes collected from a single fish. PCR reactions using the same Vβ-Jβ primer pairs previously tested with oocyte DNA confirmed that rearranged VDJ genes for all five Vβ families and Vα are present in erythrocyte DNA (Supplemental Table 3).

Identical CDR3 sequences for three clonotypes were amplified from oocyte and erythrocyte DNAs. For Vβ1–Jβ13 the identical CDR3 sequence for clonotype CAASQSGRTQASQAYF was amplified from DNA isolated from erythrocytes and oocytes. The same CDR3 sequence for clonotype CAAQGGGANQAYF was generated from erythrocyte DNA isolated from two fish with Vβ3–Jβ15 primers and was identical to that amplified with the same primers from oocyte DNA of a third fish. Vβ4–Jβ18 primers amplified an identical CDR3 sequence for clonotype CAVREFSGGREAYF from erythrocyte DNA isolated from two fish that again matched that obtained from oocyte DNA of a third fish. Thus, identical CDR3 sequences for three Vβ genes were amplified from oocyte and erythrocyte DNA. Primers for the TS.32.34 Vα-Jα sequence amplified a PCR product from erythrocyte DNA with 100% similarity to the TS.32.34 Vα-Jα sequence (Table 7). In addition, the primer combination for Vβ2 and the CDR3 sequence for clonotype CAAIMGGTQPAYF was tested using DNA isolated from erythrocytes of two fish and the sequence of the amplified PCR products was identical to that from oocytes. These results confirm those obtained using DNA from oocytes. They demonstrate that rearranged TCR genes for all five Vβ families, Vα1, and public clonotypes are integrated into germline DNA.

**Table 7:** Clonotypes of integrated TCR V to J genes in genomic DNA isolated from erythrocytes: five Vβ gene families and Vα1.

**Vβ - Jβ sequence #**	**CDR3 clonotype**	**Primers**	**Fish ID number‡**
Vβ1-Jβ13^*^	CAASQSGRTQASQAYF	Vβ1-Jβ18	51
Vβ1-Jβ20	CAASQSGEGG	Vβ1-Jβ18	52
Vβ2-Jβ24	CAARMQGDTQPAYF	Vβ2- Jβ24	7
Vβ3-Jβ17+^*^	CAAQGGGANQAYF	Vβ3-Jβ15	51
Vβ3-Jβ17+^*^	CAAQGGGANQAYF	Vβ3-Jβ15	52
Vβ4-Jβ20+^*^	CAVREFSGGREAYF	Vβ4-Jβ18	51
Vβ4-Jβ20+^*^	CAVREFSGGREAYF	Vβ4-Jβ18	52
Vβ5-Jβ27	CVADRGGSLQAYF	Vβ5-Jβ27	7
Vβ5 like-Jβ20	CAAYYHRVGGREAYF	Vβ5-Jβ15	7
Vα1	CALVPTTGS	VαTS.32.34 Jα TS.32.34	51
Vα1	CALVPTTGS	VαTS.32.34 Jα TS.32.34	52

# *Sequence data shown in Supplementary Information*.

* *Same CDR3 sequence in oocyte*.

+ *Identical sequence*.

‡ *Individual fish identification number*.

### Vβ CDR3 frameshift sequences

Five Vβ-Jβ sequences amplified from oocyte DNAs had a frameshift or stop codon in the CDR3 sequence. In each case the frameshift or stop codon was located in the Dβ-Jβ junction sequence. A Vβ1–Jβ18 primer pair amplified a TB4–Jβ17 sequence, which had had five nucleotides added at the Vβ-Dβ junction and a deletion of one nucleotide at the 5′ end of the Dβ gene. The sequence of the Dβ-Jβ junction could result from deletion of three nucleotides at the 3′ end of the Dβ gene and five nucleotides at the 5′ end of the Jβ17 gene or, alternatively, from deletion of one nucleotide at the 3′ end of the Dβ gene and seven nucleotides at the 5′ end of the Jβ17 gene. In either case, this resulted in a frameshift at the Dβ-Jβ junction. The PCR products amplified from two fish using a Vβ2–Jβ24 primer pair had different CDR3 sequences. In both fish there were additions of nucleotides at the Vβ-Dβ junction, deletion of nucleotides from both the 5′ and 3′ ends of the Dβ gene and addition of nucleotides at the Dβ-Jβ junction. No nucleotides were deleted from the 5′ Jβ gene sequence. In both fish this resulted in formation of a TAA stop codon at the Dβ-Jβ junction. In two fish an identical CDR3 sequence was amplified with a Vβ3–Jβ15 primer pair, which had eleven nucleotides added at the Vβ-Dβ junction, and deletions of one nucleotide at the 5′ and four nucleotides at the 3′ end of the Dβ gene and a single nucleotide from the 5′ end of the Jβ17 gene. Three nucleotides were added at the Dβ-Jβ junction. This resulted in a frameshift at the Dβ-Jβ17 junction. Consequently, rearranged Vβ-Jβ sequences containing frameshifts or stop codons are also integrated into the germline genome (Table 8, Supplemental Table 4).

**Table 8:** Germline integrated TCR Vβ - Jβ genes with stop codons or frameshifts in CDR3 sequence.

**Vβ gene**	**CDR3 sequence #**
TB4-Jβ17^+^ Frame-shift Fish 3	TB4 Dβ Jβ17 **G**GGACAGGGGG**C** TGTGCA**GCCAAT CCTCT**GGTGGTGCCAATCAAGCTTACTTT TGTGCA***ATCTT***GGACAGGGGGTGGTGCCAATCAAGCTTACTTT C A I L D R
Vβ2-Jβ29 Stop Codon Fish H6	Vβ2 Dβ Jβ29 **GGGAC**AGGG**GGC** TGTGCAGCCA**GA** AACAACCAGCCTGCATACTTT TGTGCAGCCA***AAG***AGGG***T**T*AACAACCAGCCTGCATACTTT C A A K E G
Vβ2-Jβ24 Stop Codon Fish 9	Vβ2 Dβ Jβ24 **G**GGACAG**GGGGC** TGTGCAGCCAGA AACACTCAGCCTGCATACTTC TGTGCAGCCAGA***CC***GGACAG***A**T*AACAC*T*CAGCCTGCATACTTC C A A R P D R
Vβ3-Jβ17^*^ Frame-shift Fish 3	Vβ3 Dβ Jβ17 **G**GGACAGG**GGGC** TGTG**TAACC C**CTCTGGTGGTGCCAATCAAGCTTACTTT TGTG***CAGCACGGATA***GGACAGG***CG**GCT*CTGGTGGTGCCAATCAAGCTTACTTT C A A R I G Q A
Vβ3-Jβ17^*^ Frame-shift Fish 9	Vβ3 Dβ Jβ17 **G**GGACAGG**GGGC** TGTG**TAACC C**CTCTGGTGGTGCCAATCAAGCTTACTTT TGTG***CAGCACGGATA***GGACAGG***CG**GCT*CTGGTGGTGCCAATCAAGCTTACTTT C A A R I G Q A

*The nucleotide sequences are shown for the CDR3 of rearranged, germline integrated genes with stop codons or frameshifts at the Dβ - Jβ junction. For comparison the cDNA sequences of Vβ genes, and genomic sequences for Dβ and Jβ genes are also shown. Predicted aa sequences preceding the stop codons or frameshifts are presented. The nucleotides that appear to be deleted are shown in bold. The nucleotides apparently added at Vβ - Dβ or Dβ - Jβ junctions are shown in bold italics. Frameshift or stop codons are underlined*.

# Complete sequence data shown in Supplementary Information.

+ *Only single stranded sequence*.

* *Identical sequence*.

### Germline rearranged Vβ to Jβ sequences contain an intron

**Figure 4:**
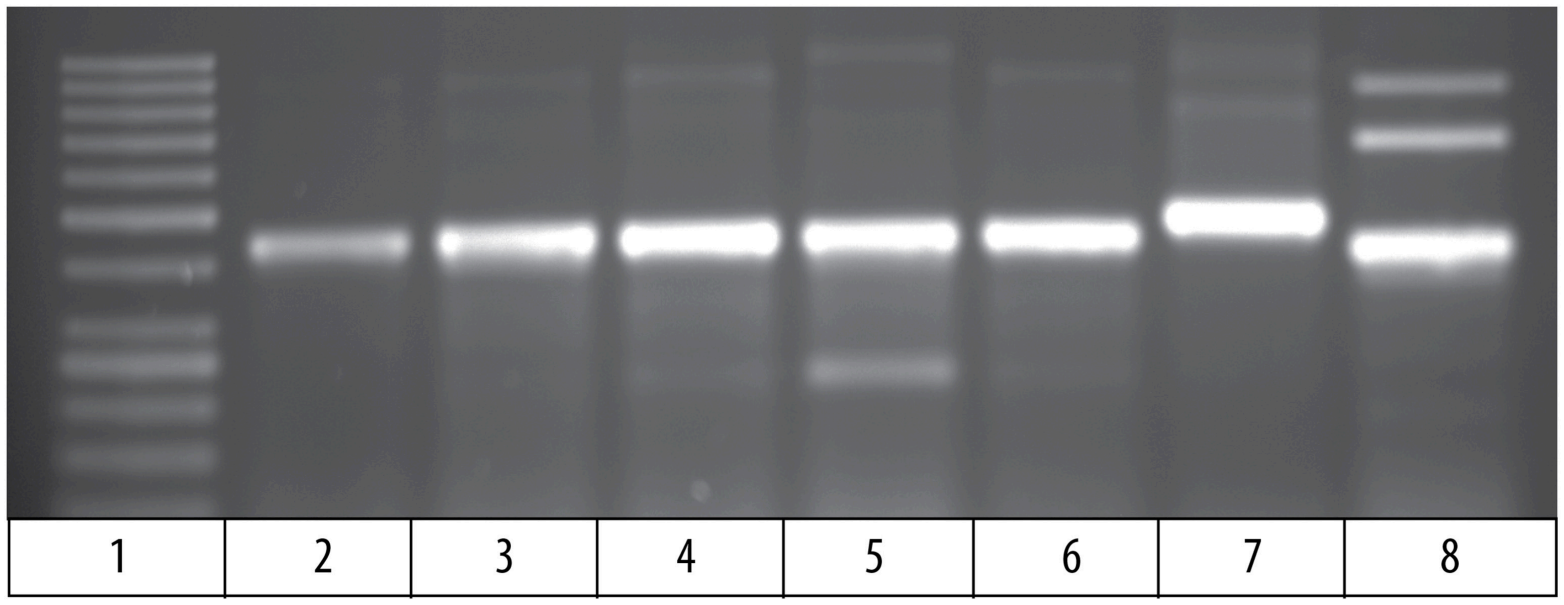
PCR products amplified from oocyte DNA using primer pairs for the leader sequences of Vβ genes 2, 4 and 5 (Table 4) and CDR3 sequences (Table 3). The Vβ2 CDR3 sequences are public clonotypes and the Vβ4 and Vβ5 CDR3 sequences are clonotypes of rearranged VDJ genes in the germline. (1) GeneRuler low range ladder (Thermo-Fisher), (2) Vβ2 to clonotype CAAHRGANPAYF, (3) Vβ2 to clonotype CAARKAYGNNPAYF, (4) Vβ2 to clonotype CAARQLTNTYPAYF, (5) Vβ2 to clonotype CAAKDRGLSSPAYF, (6) Vβ2 to clonotype CAAKQISGVNPAYF, (7) Vβ4 to CDR3 primer (Table 4), (8) Vβ5 to CDR3 primer (Table 4).

Comparison of the cDNA sequence for each of the five Vβ genes with their genomic sequence on chromosome 9 showed that introns, ranging in size from 100 to 149 bp, are present between the leader and Vβ gene sequences for each of the five Vβ genes. To determine if the rearranged VDJ sequences in the germline also contain these introns, we designed PCR primers for the CDR3 sequences of rearranged VDJ genes for Vβ1 through Vβ5. We used these CDR3 primers and a primer for the corresponding Vβ leader sequence to amplify PCR products from oocyte DNA. This resulted in amplification of products from oocyte DNA of 454 bp for Vβ2, 495 bp for Vβ4, and 446 bp for Vβ5, which correspond to the lengths predicted if introns were present between the leader and CDR3 sequences in the germline rearranged genes. In contrast, bands of 349 bp for Vβ2, 346 bp for Vβ4, and 346 bp for Vβ5 sequences corresponding to germline rearranged genes lacking an intron were not observed (Figure 4). Although bands of the expected size were observed for Vβ1 and Vβ3, multiple additional bands were also observed (not shown).

The 495 bp Vβ4 PCR products amplified from oocyte DNAs of two fish were isolated and sequenced. The combined leader, intron, and Vβ4 gene sequences had 98 % identity to the Vβ4 genome sequences. This confirmed that an intron is present in the rearranged germline sequence of a Vβ4 gene (Supplemental Table 5). For Vβ2, a primer for the Vβ2 leader was paired with primers for the CDR3 sequence of five public clonotypes, which resulted in amplification of 450 bp products for all clonotypes. The sequences of the PCR products for all five clonotypes had ~98% identity to the Vβ2 sequence and at their juncture with the 5′ Vβ2 sequence contained the sequence 5′ TTTTTCATCA 3′, which is identical to the 3′ terminus of the Vβ2 intron sequence. For four clonotypes the sequences 5′ of the 5′ TTTTTCATCA 3′ intron sequence did not match the upstream Vβ2 intron sequence. However, for clonotype CAAHRGANPAYF the intron sequence was 89 % similar with that of the genomic Vβ2 intron sequence (Supplemental Table 5). The sequence results confirm that public Vβ2 clonotypes have an intron sequence 5′ of the Vβ2 gene sequence, and the intron sequence for clonotype CAAHRGANPAYF is highly similar to that of the Vβ2 genomic sequence. These results demonstrate that rearranged VDJ sequences in the germline for Vβ2 and Vβ4, have an intron between the leader and Vβ gene sequences. The presence of introns in the rearranged VDJ sequences in the germline suggest that a DNA transposon, such as Tc1/mariner, mediated their transposition into the germline.

### Proximity of rearranged VDJβ genes to Cβ1

We used primers for Vβ2 and Vβ4 leader sequences in combination with a primer for Cβ1 to assess the proximity of rearranged VDJβ genes to the Cβ1 gene. PCR products of less than, or equal to, 5 kb were not detected. This demonstrates that the rearranged VDJβ genes are not closely linked to a Cβ1 gene in the germline genome. It suggests that somatic recombination is required to bring the rearranged VDJβ genes into proximity of a Cβ1 gene to enable expression.

### Vα genes have integrated Vα to Jα copies

The αβ TCR is a heterodimeric protein formed by pairing of a TCR α chain with a TCR β chain. To determine if rearranged sequences for Vα and Jα genes are also found in the germline genome we tested Vα and Jα primer combinations to determine if a Vα-Jα sequence could be amplified by PCR from oocyte DNA. TS.32.34 is a cytotoxic T cell line cloned from PBL isolated from a channel catfish. The TS.32.34 Vα-Jα sequence was determined by sequencing a cloned cDNA generated from RNA isolated from the cell line (25). Primers based on the TS.32.34 Vα-Jα sequence resulted in amplification of a 210 bp PCR product from oocyte DNA of three fish, which had 100% similarity with the TS.32.34 Vα-Jα sequence. As the oocyte sequence is identical to the sequence cloned from this cell line, this confirmed that the germline-integrated, rearranged Vα-Jα genes are expressed in circulating αβ T cells (Table 6 and Supplementary Tables 2, 3).

## Discussion

In cartilaginous fish the genes coding for IgH and IgL chains of the BCR are arranged in a cluster configuration with a single copy of a V, (D), J and C gene in each cluster and multiple clusters arranged in tandem in the genome. In some sharks, the IgH V, D and J gene segments within a cluster can be organized as separate genes, or in fused VD-J or VDJ configurations. Similarly, IgL V and J genes segments are arranged as both separate V and J gene segments, or in a fused VJ configuration. The rearrangement to a fused configuration affects only those genes within a cluster and their origin is attributed to RAG activity in germ cells (20, 21). In bony fishes (teleosts), IgL genes are also organized in a cluster configuration with V, J, and C gene segments within a single cluster, but V and J genes are not found in a fused configuration (19).

The possibility that rearranged, fused TCRβ VDJ gene sequences are stably integrated into the *I. punctatus* genome was suggested by characterization of expressed TCR Vβ2 CDR3 repertoires in four channel catfish during infection with *I. multifiliis*. This showed that in each fish a single DNA sequence in combination with the same J gene was preferentially used to code for the CDR3 sequence of each public Vβ2 clonotype. The identical CDR3 DNA sequence was expressed by all four fish (15). This possibility was confirmed by using PCR to amplify rearranged TCRβ Vβ2 sequences coding for these expressed public clonotypes from germline DNA, isolated from oocytes. Sequencing the PCR products demonstrated that the CDR3 sequences amplified from genomic DNA matched their respective cDNA sequences. This analysis was expanded to show that rearranged sequences for a Vα1–Jα gene and Vβ-Jβ genes for all five Vβ gene families are integrated into the germline genome. These results were confirmed using DNA isolated from erythrocytes, which were collected by laser dissection microscopy from blood smears to insure that they were not contaminated with T cells. Three identical clonotypes were amplified using DNA from oocytes or erythrocytes of different fish, providing further support that these are germline integrated sequences. A single fused IgH gene was previously identified in the channel catfish genome (17, 18). Our preliminary data also indicated that copies of rearranged, fused V(D)J sequences for IgH as well as IgL genes are integrated into the germline genome of channel catfish (not shown).

The organization of these rearranged TCR V(D)J gene sequences in diploid cells and how this affects the processes that control allelic exclusion are not known. Not all combinations of Vβ-Jβ primers resulted in amplification of PCR products. Thus, it is not clear how many different rearranged Vβ-Jβ sequences are integrated into the germline genome or if multiple copies of the same sequence are integrated. Only rearranged Vβ-Jβ sequences appear to be integrated into the germline genome, as rearranged VDJ to Cβ1 sequences were not detected. In all cases the CDR3 sequences showed random deletion and addition of nucleotides at Vβ-Dβ and Dβ-Jβ junctions. Consequently, these germline integrated, rearranged Vβ-Dβ-Jβ genes are most likely the products of the RAG-mediated somatic recombination of Vβ, Dβ, and Jβ genes and TdT modification of Vβ-Dβ and Dβ-Jβ junctions that occur during the development of αβ T cells.

### Genome organization

The *I. punctatus* genome has been sequenced to ~97% completeness, but the projected sizes of the two reference genomes range between 0.84 Gb and 1.01 Gb (10, 11). Estimates of repetitive sequences range between 275 Mb and 418 Mb, or 33 % to 44 % of the genome (11, 27). The Tc1-mariner family DNA transposon is the most abundant class of TEs and accounts for ~9 % of the genome (11, 27, 28). Sequences corresponding to rearranged Vβ-Jβ or Vα-Jα genes were not detected by BLAST searches of the two *I. punctatus* reference genomes. As there are multiple copies of rearranged Vβ-Jβ gene sequences for each of the five Vβ gene families, which differ in sequence by only a few nucleotides in the CDR3 and J genes, they constitute families of highly repetitive sequences. Their absence in the reference genomes may be due to the technical difficulties associated with sequencing and assembly of repetitive sequences (29, 30).

The organization of rearranged Vβ-Jβ and Vα-Jα genes in the germline genome remains to be determined, but they must be located on chromosomes that also incorporate TCR C gene(s) to permit transcription of mRNAs coding for full length TCRα and TCRβ chains. A Vα1–Jα sequence is expressed by the cloned channel catfish cytotoxic T cell line TS.32.34, and a rearranged VJ sequence for this Vα1–Jα sequence is found in the germline genome. This confirms that a germline-rearranged TCRα gene is expressed in an αβ T cell. The rearranged Vβ-Jβ genes must lie at some distance from the TCR Cβ1 and Cβ2 genes because they are not found in ~16 kb of DNA upstream of TCR Cβ1 gene in a lambda clone (9). It is possible that they are highly dispersed, like the five Vβ genes, which are arranged over 204 kb of chromosome 9, and that somatic recombination brings the rearranged V(D)J genes into proximity of a C gene, which enables transcription of full length mRNAs. The sequences flanking the rearranged Vβ-Jβ and Vα-Jα genes are of special interest, as these may provide insight into the origin of the rearranged V(D)J genes and their evolutionary history.

The causal mechanisms that lead to the dominance of copies of transcripts from the germline rearranged genes are not understood. Following infection, the abundance of copies of the dominant CDR3 sequence that codes for a public clonotype overshadows the multiple, but much less abundant, other CDR3 sequences that code for the same clonotype. On average 22 other CDR3 sequences coded for each public clonotype, but together these comprised only ~3% of copies of sequences coding for a public clonotype (15). This suggests that two classes of αβ T cells, which express CDR3 sequences coding for the same clonotype, may potentially respond to a primary infection. The first class is comprised of a population of αβ T cells, generated by classical V(D)J rearrangements, which express CDR3 sequences coding for the same clonotype, but these αβ T cell clones are present in low abundance. The second class expresses the dominant CDR3 sequence from the germline rearranged gene. This class of αβ T cells undergoes a more rapid clonal expansion when encountering antigen recognized by its TCR.

### Origin of germline sequences

Transposable elements are predicted to have played a crucial role in the evolution of the vertebrate adaptive immune system, as the random insertion of a TE into a gene coding for a precursor of the TCR is hypothesized to have given rise to the families of genes that code for the TCR in today's vertebrates. The TE was probably a “RAG transposon” composed of RAG genes and flanked by terminal inverted repeats (1, 31–37). In teleosts and most mammals the genes coding for the αβ TCR are organized on a chromosome in a translocon configuration in which the V gene families are grouped together at some distance from the J gene families. During development of αβ T cells, somatic rearrangement of a Dβ gene with a Jβ gene is followed by rearrangement of a Vβ gene to the fused DβJβ gene. This results in deletion of all sequences in the chromosome located between the rearranging Vβ and DβJβ genes (1, 26, 32). Thus, rearrangement of the most distal Vβ gene deletes all other Vβ genes and potentially many Jβ genes. This differs from a cluster configuration in which only those genes in each cluster undergo rearrangement. Consequently, it is unlikely in channel catfish that these germline rearranged TCR genes are the result of RAG activity in germ cells, as proposed to occur in some sharks (20, 21). Instead, we suggest that these germline integrated copies of rearranged TCR genes originated by conventional RAG mediated rearrangement of V(D)J genes during development of αβ T cells that subsequently underwent TE transposition to germline DNA (38). As the rearranged genes retain their introns splitting the leader and V gene sequences, this suggests that a DNA transposon mediated their transposition into the germline (22). Only rearranged V(D)J gene sequences are apparently integrated into the germline genome, which raises the possibility that the enzymatic steps involved in the rearrangement of V gene to a (D)J gene and TE transposition may be linked. TEs are abundant in the channel catfish genome with the DNA-based TE, Tc1/mariner comprising 9% of the genome (11, 27, 28). The Tol2 TE has been shown to be active in fertilized zebrafish eggs and leads to Tol2 transposition into the germline (39). These TE mediated transpositions must have occurred multiple times, as rearranged V(D)J sequences for all five Vβ families and Vα1 are found in the germline.

### Evolutionary benefit

These results reveal a new dimension to the adaptive immune system of vertebrates. In channel catfish rearranged TCRα and TCRβ sequences are present in the germline genome and in response to *I. multifiliis* infection transcripts from these rearranged TCR germline sequences dominate the repertoire of expressed Vβ2 sequences coding for public clonotypes. However, rearranged Vβ2 genes in the germline with CDR3 sequences coding for clonotypes that were not present in the repertoires expressed during *I. multifiliis* infection were also identified. In sharks, the fused Ig genes are proposed to protect against infection by common pathogens, but are only expressed early in development (21). That pattern of expression differs from channel catfish in which the rearranged Vβ2 genes are expressed in mature fish.

Teleosts are ectotherms and cold water temperatures could potentially slow the engagement of their adaptive immune system with an infecting pathogen. Teleosts also lack lymph nodes, which may reduce the efficiency of interactions between αβ T cells and antigen presenting cells (40). Acting together, the combination of a slowed response in cold conditions and lack of lymph nodes could potentially reduce the protection afforded by the adaptive immune system. In addition, only a small fraction of the potential CDR3 repertoire is expressed by αβ T cells circulating in peripheral blood at any one point in time (2, 5). All of these effects could be offset by having an array of germline integrated, rearranged TCRs, expressed by circulating αβ T cells, which code for clonotypes that recognize antigens from commonly encountered pathogens, such as *I. multifiliis*. Engagement of any of these germline-coded clonotypes with antigen would result in immediate and preferential expansion of the αβ T cells expressing the functional clonotype, and more rapid mobilization of populations of αβ T cells that recognize and bind antigens expressed by a pathogen. This pattern was observed following infection of channel catfish with *I. multifiliis*, in which TCRs expressed by germline integrated, rearranged Vβ2 TCRs dominated the expressed repertoires. The germline integrated, rearranged TCRs appear to function as an evolutionarily conserved, repertoire of CDR3 sequences.

## Author contributions

RF and RS designed the experiments. RS and AC performed the experiments. RF, FN, RS, and HD analyzed the experiments. RF and HD wrote the manuscript.

### Conflict of interest statement

The authors declare that the research was conducted in the absence of any commercial or financial relationships that could be construed as a potential conflict of interest.

## Abbreviations

aa: amino acid
BCR: B cell receptor
TCR: T cell receptor
CDR3: complementarity determining region 3
V: TCR variable gene
D: TCR diversity gene
J: TCR joining gene
C: TCR constant gene.
