# Supplementary material for: Rearranged T Cell Receptor Sequences in the Germline Genome of Channel Catfish Are Preferentially Expressed in Response to Infection
Source: Front Immunol. 2018 Sep 27;9:2117. doi: 10.3389/fimmu.2018.02117 (PMC6170632; doi:10.3389/fimmu.2018.02117)
Supplement: Supplementary file 1 [file Table_1.DOCX]

Rearranged T Cell Receptor Sequences in the Germline Genome of Channel Catfish are Preferentially Expressed in Response to Infection

R. Craig Findly^a1^, Frank D. Niagro^a^, Ryan P. Sweeney^a^, Alvin C. Camus^b^ and Harry W. Dickerson^a^

^1^Corresponding author:

R. Craig Findly

Department of Infectious Diseases,

College of Veterinary Medicine,

University of Georgia

Athens, GA 30602

Phone: (706) 542-5793

Email: [rfindly@uga.edu](mailto:rfindly@uga.edu)

**Supplementary Material**

Supplementary Table 1. Channel catfish TCRβ Vβ2 to CDR3 sequences for six public clonotypes amplified by PCR from DNA isolated from oocytes or erythrocytes. The fish from which the oocytes or erythrocytes were isolated are shown.

**1. CAAHRGANPAYF**

Fish 1

AACAGTAACACAGTTATGGCACTGATTGGATATACCTATACGGCTACGAGTAAGCCAGAGTACGAGGACGGATTTAATGATAGGTACAAACAGAGCAGAAAGAGCATAACTGAAGGAAGTCTGACCATCTCTAAACTCCTCCAGTCAGACTCGGCTGTTTATTACTGTGCAGCCCACAGG

Fish 9

AACAGTAACACAGTTATGGCACTGATTGGATATACCTATACGGCTACGAGTAAGCCAGAGTACGAGGACGGATTTAATGATAGGTACAAACAGAGCAGAAAGAGCATAACTGAAGGAAGTCTGACCATCTCTAAACTCCTCCAGTCAGACTCGGCTGTTTATTACTGTGCAGCCCACAGG

Fish H1

AACACAGTTATGGCACTGATTGGATATACCTATACGGCTACGAGTAAGCCAGAGTACGAGGACGGATTTAATGTTAGGTACAAACAGAGCAGAAAGAGCATAACTGAAGGAAGTCTGACCATCTCTAAACTCCTCCAGTCAGACTCGGCTGTTTATTACTGTGCAGCCCACAGG

**2. CAAIMGGTQPAYF**

Fish 1

GAGTACGAGGACGGATTTAATGTTAGGTACAAACAGAGCAGAAAGAGCATAACTGAAGGAAGTCTGACCATCTCTAAACTCCTCCAGTCAGACTCGGCTGTTTATTACTGTGCAGCCATAATGGGTGGCACTCAG

Fish 9

GAGTACGAGGACGGATTTAATGTTAGGTACAAACAGAGCAGAAAGAGCNNNNNNNAAGGAAGTCTGACCATCTCTAAACTCCTCCAGTCAGACTCGGCTGTTTATTACTGTGCAGCCATAATGGGTGGCACTCAGC

Fish H1

CAGTTATGGCACTGATTGGATATACCTATACGGCTANNNNNNNNNNNGNGTACGAGGACGGATTTAATGTTAGGTACAAACAGAGCAGAAAGAGCATAACTGAAGGAAGTCTGACCATCTCTAAACTCCTCCAGTCNNACTCGGCTGTTTATTACTGTGCAGCCATAATGGGTGGCACTCAG

Fish 6

GAGTACGAGGACGGATTTAATGNTAGGTACAAACAGAGCAGAAAGAGCATAACTGAAGGAAGTCTGACCATCTCTAAACTCCTCCAGTCAGACTCGGCTGTTTATTACTGTGCAGCCATAATGGGTGGCACTCAG

Fish H1 Erythrocyte

GTTATGGCACTGATTGGATATACCTATACGGCTACGAGTAAGCCAGAGTACGAGGACGGATTTAATGTTAGGTACAAACAGAGCAGAAAGAGCATAACTGAAGGAAGTCTGACCATCTCTAAACTCCTCCAGTCAGACTCGGCTGTTTATTACTGTGCAGCCATAATGGGTGGCACTCAGC

Fish D1 Erythrocyte

ACAGTAACACAGTTATGGCACTGATTGGATATACCTATACGGCTATGAGTAAGCCAGAGTACGAGGACGGATTTAATGTTAGGTACAAACAGAGCAGAAAGAGCATAACTGAAGGAAGTCTGACCATCTCTAAACTCCTCCAGTCAGACTCGGCTGTTTATTACTGTGCAGCCATAATGGGTGGCACTCAGC

**3. CAAKDRGLSSPAYF**

Fish 1 AACAGTAACACAGTTATGGCACTGATTGGATATACCTATACGGCTACGAGTAAGCCAGAGTACGAGGACGGATTTAATGATAGGTACAAACAGAGCAGAAAGAGCATAACTGAAGGAAGTCTGACCATCTCTAAACTCCTCCAGTCAGACTCGGCTGTTTATTACTGTGCAGCCAAAGACAGG

Fish 9

AACAGTAACACAGTTATGGCACTGATTGGATATACCTATACGGCTACGAGTAAGCCAGAGTACGAGGACGGATTTAATGATAGGTACAAACAGAGCAGAAAGAGCATAACTGAAGGAAGTCTGACCATCTCTAAACTCCTCCAGTCAGACTCGGCTGTTTATTACTGTGCAGCCAAAGACAGG

Fish H1

AACACAGTTATGGCACTGATTGGATATACCTATACGGCTACGAGNNNNCCAGAGTACGAGGACGGATTTAATGTTAGGTACAAACAGAGCAGAAAGAGCATAACTGAAGGAAGTCTGACCATCTCTAAACTCCTCCAGTCAGACTCGGCTGTTTATTACTGTGCAGCCAAAGACAGG

**4. CAARKAYGNNPAYF**

Fish 1

CGGATTTAATGATAGGTACAAACAGAGCAGAAAGAGCNNNNCTGAAGGAAGTCTGACCATCTCTAAACTCCTCCAGTCAGACTCGGCTGTTTATTACTGTGCAGCCAGAAAAGC

Fish 9

GAAGGAAGTCTGACCATCTCTAAACTCCTCCAGTCAGACTCGGCTGTTTATTACTGTGCAGCCAGAAAAGC

Fish H1

TCCTCCAGNNNNNCTCNNNTGNNTATTACTGTGCAGCCAGAAAA

Fish 6

AGCCAGAGTACGAGGACGGATTTAATGNTAGGTACAAACAGAGCAGAAAGAGCATAACTGAAGGAAGTCTGACCATCTCTAAACTCCTCCAGTCAGACTCGGCTGTTTATTACTGTGCAGCCAGAAAAGC

**5. CAARKDKYEAYF**

Fish 1

CAGTTATGGCACTGATTGGATATACCTATACGGCTACGNNNNNNNNNGAGTACGAGGACGGATTTAATGTTAGGTACAAACAGAGCAGAAAGAGCATAACTGAAGGAAGTCTGACCATCTCTAAACTCCTCCAGTCAGACTCGGCTGTTTATTACTGTGCAGCCAGAAAAGACAA

Fish 9

AACAGTAACACAGTTATGGCACTGATTGGATATACCTATACGGCTACGAGTAAGCCAGAGTACGAGGACGGATTTAATGATAGGTACAAACAGAGCAGAAAGAGCATAACTGAAGGAAGTCTGACCATCTCTAAACTCCTCCAGTCAGACTCGGCTGTTTATTACTGTGCAGCCAGAAAAGACAA

H1

GTTATGGCACTGATTGGATATACCTATACGGCTACGNGNNNNCCNGAGTACGAGGACGGATTTAATGTTAGGTACAAACAGAGCAGAAAGAGCATAACTGAAGGAAGTCTGACCATCTCTAAACTCCTCCAGTCAGACTCGGCTGTTTATTACTGTGCAGCCAGAAAAGACAA

**6. CAARQLTNTYPAYF**

Fish 1

AACAGTAACACAGTTATGGCACTGATTGGATATACCTATACGGCTACGNGNNNGCCAGAGTACGAGGACGGATTTAATGATAGGTACAAACAGAGCAGAAAGAGCATAACTGAAGGAAGTCTGACCATCTCTAAACTCCTCCAGTCAGACTCGGCTGTTTATTACTGTGCAGCCAGACAGCTAAC

Fish 9

CAGTAACACAGTTATGGCACTGATTGGATATACCTATACGGCTACGAGTAAGCCAGAGTACGAGGACGGATTTAATGATAGGTACAAACAGAGCAGAAAGAGCATAACTGAAGGAAGTCTGACCATCTCTAAACTCCTCCAGTCAGACTCGGCTGTTTATTACTGTGCAGCCAGACAGCTAAC

Fish H1

AACAGTAACNCAGTTATGGCACTGATTGGATATACCTATACGGCTACGAGNNANCCAGAGTACGAGGACGGATTTAATGTTAGGTACAAACAGAGCAGAAAGAGCATAACTGAAGGAAGTCTGACCATCTCTAAACTCCTCCAGTCAGACTCGGCTGTTTATTACTGTGCAGCCAGACAGCTAAC
