# Supplementary material for: Rearranged T Cell Receptor Sequences in the Germline Genome of Channel Catfish Are Preferentially Expressed in Response to Infection
Source: Front Immunol. 2018 Sep 27;9:2117. doi: 10.3389/fimmu.2018.02117 (PMC6170632; doi:10.3389/fimmu.2018.02117)
Supplement: Supplementary file 2 [file Table_2.DOCX]

Supplemental Table 2. Channel catfish TCR Vβ to Jβ gene and Vα to Jα gene sequences amplified by PCR from DNA isolated from oocytes. Predicted CDR3 aa sequences are underlined. The fish from which the oocytes were isolated are shown.

**TCRβ**

Fish 8 Vβ1–Jβ13

GGTATCGGCAAAACAGTCGTACAGTTCTGGCACTGATCGGATATACCATGACAGCTAAAAGTGATCCAAAATATTANNNNNNATTTNNNGGTTNNTNCNCCCNGAGCAGACAGGGCACACTTGCAGGAACTCTGACCATCTCTAATCTCCGCCAGTCAGACTCTGCTGTTTATTACTGTGCAGCCAGTCCCGGCGGTGCCAGTCAAGCTTACTTTGGTGGAGGAACCAAGTTAACAGT

Translation

YRQNSRTVLALIGYTMTAKSDPKY???F?G????SRQGTLAGTLTISNLRQSDSAVYYCAASPGGASQAYFGGGTKLT

Fish 9 Vβ1–Jβ13

GGTATCGGCAAAACAGTCGTACAGTTCTGGCACTGATCGGATATACCATGACAGCTATTANTTTTNCAAAATAGGTNNNNNAATGGGACGATCGGTTCACCCTGAGCAGTCAGAGCACACTTGCAGGAACTCTGACCATCTCTAATCTCCGCCAGTCAGACTCTGCTGTTTATTACTGTGCAGCCAGTCAATCAGGNAGGACTCAGGCCAGTCAAGCTTACTTCGGTGGAGGAACCAAGTTAACAG

Translation

YRQNSRTVLALIGYTMTAI?F?K*???WDDRFTLSSQSTLAGTLTISNLRQSDSAVYYCAASQSGRTQASQAYFGGGTKLT

Fish 3 Tβ14–Jβ21

ATCGGCAAAACAGTCGTACTGTTATGGCACTAATTGGATATACTGCTACAGCGTCGGGTGATCCAAACTATGAGGATGGATTTAAAGATGGGTTCAAACAGAGCAGGCAGGGCACACTTAATGGAAGCCTGACCATCTCTAATCTCCGCCAGTCAGACTCTGCTGTTTATTACTGTGCAGCCAGTATAGATGGAAACAATCCAGCTTACTTTGGTGAAGGGACCAAACTAACCGTTCTTG

Translation

RQNSRTVMALIGYTATASGDPNYEDGFKDGFKQSRQGTLNGSLTISNLRQSDSAVYYCAASIDGNNPAYFGEGTKLTVL

Fish 8 Vβ2–Jβ27

TTCCAGTGCNNNNACAATGACAATACCTTACAAACAATGTTATGATACCTGCAAAACAGTAACACAGTTATGGCACTGATTGGATATACCTATACGGCTATGAGTAAGCCAGAGTACGAGGACGGATTTAATGATAGGTACAAACAGAGCAGAAAGAGCATAACTGAAGGAAGTCTGACCATCTCTAAACTCCTCCAGTCAGACTCGGCTGTTTATTACTGTGCAGCGAAAATAACAGGGGATTATAGCCTTCAAGCTTACTTTGGTGGAGGAACCAAGNTAACAGTTCA

Translation

QC??NDNTLQTML*YLQNSNTVMALIGYTYTAMSKPEYEDGFNDRYKQSRKSITEGSLTISKLLQSDSAVYYCAAKITGDYSLQAYFGGGTK?TV

Fish 8 Vβ2–Jβ13

TTCCAGTGCNNNNNNAATGACAATACCTTACAAACAATGTTATGGTACCTGCAAAACAGTAACACAGTTATGGCACTGATTGGATATACCTATACGGCTACGAGTAAGCCAGAGTACGAGGACGGATTTAATGTTAGGTACAAACAGAGCAGAAAGAGCATAACTGAAGGAAGTCTGACCATCTCTAAACTCCTCCAGTCAGACTCGGCTGTTTATTACTGTGCAGCCAGAACGGGGATCTCTGGCGGTGCCAGTCAAGCTTACTTTGGTGGAGGAAC

Translation

FQC??NDNTLQTMLWYLQNSNTVMALIGYTYTATSKPEYEDGFNVRYKQSRKSITEGSLTISKLLQSDSAVYYCAARTGISGGASQAYFGGG

Fish H6 Vβ3–Jβ17

ACTGAGCCAAAGAACGAGGAAGATTTTAAAGATCGGTTCGAACAGAGCAGACAGAGCATAATGGCAGGAAAACTTACCATCTCTAAAGTACTTCAGTCAGACTCTGCTGTTTATTACTGTGCAGCACGTGATGGGCAGGGGATTGGTGCCAATCAAGCTTACTTTGGTGGAGGAACCAAGTTAACAG

Translation

TEPKNEEDFKDRFEQSRQSIMAGKLTISKVLQSDSAVYYCAARDGQGIGANQAYFGGGTKLT

Fish 9 Vβ3–Jβ17

GCAGACAGATCATAATGGCAGGAAAACTTACCATCTCTAAAGTACTTCAGTCAGACTCTGCTGTTTATTACTGTGCAGCACAGGGTGGTGGTGCCAATCAAGCTTACTTTGGTGAAGGGACCAAGTTAACAGTA

Translation

RQIIMAGKLTISKVLQSDSAVYYCAAQGGGANQAYFGEGTKLTV

Fish 3 Vβ4–Jβ20

GACTTTGGAAAATCCGACCAAAAGAAATTTTCAGCTATTAAAACAGTTCCTGAGAACGGCTCATTCACAGTGAAAGACGCGGATTATAATGACAACGCTGTGTATTTCTGTGCCGTGAGAGAATACAATGGGGGCCGTGAAGCTTACTTTGGTGGAGGAACCAAGTTAACAGTT

Translation

DFGKSDQKKFSAIKTVPENGSFTVKDADYNDNAVYFCAVREYNGGREAYFGGGTKLTV

Fish H7 Vβ4–Jβ20

GACTTTGGAAAATCCGACCAAAAGAAATTTTCAGCTATTAAAACAGTTCCTGAGAACGGCTCATTCACAGTGAAAGACGTGGATTATAATGACAACGCTGTGTATTTCTGTGCCGTGAGAGAATTCTCTGGGGGCCGTGAAGCTTACTTTGGTGGAGGAACCAAGTTAACAGT

Translation

DFGKSDQKKFSAIKTVPENGSFTVKDVDYNDNAVYFCAVREFSGGREAYFGGGTKLT

Fish 9 Vβ4–Jβ11

GACTTTGGAAAATCAGACCAAAAGAAATTTTCAGCTATTAAAACAGTTCCTGAGAACGGCTCCTTCACAGTGAAAGACGTGGATTATAATGACAACGCTGTGTATTTCTGTGCCGGAGAGATTGTGNCCNGTCAAGCTTANNTCNGTGGAGGAACCANGTT

Translation

DFGKSDQKKFSAIKTVPENGSFTVKDVDYNDNAVYFCAGEIV??QA???GGT?

Fish 8 Vβ5–Jβ2/3

CAGGNNCAGGATTTAAATACATGGGGTATCTGAACACTATTTTTCCAAAAGAAGAGGCGGAATTTGGAACGAAGATCAAGTTGAGTGGTGATGGGAGAAAGAGCGGCTCTATGACCATAAACAGTCTTTCCGTGAACGACAGTGCTGTATATTTCTGTGTAGCCTTCCCGGGACAGGGGTTCACTGGCTCTCAAGCATATTTTGGAGCTGGNNCAAAAC

Translation

??GFKYMGYLNTIFPKEEAEFGTKIKLSGDGRKSGSMTINSLSVNDSAVYFCVAFPGQGFTGSQAYFGA??K

Fish 8 Vβ5 like–Jβ17

CAGGANNNNNNATTTAAATACATGGGATACCTTTCAAATGCTTACCCAAAGCTAGAGGAAGGATTTGAAACCAAAATCAAACTGAGTGGTGATGGGAGCAAAAACGGTTCTTGAGCATAAAGAGTCTTTCAGTGAACGACAGTGCTGTGTATTTCTGTGCAGCTAACTCTGGTGGTGCCAATCAAGCTTACTTTGGTGAAG

Translation

Q???FKYMGYLSNAYPKLEEGFETKIKLSGDGSKNGSLSIKSLSVNDSAVYFCAANSGGANQAYFGE

**TCR Vα**

Fish 8 Vα TS32.34 - Jα TS32.34

CCTACAGGAAACTACCTGCACTGGTACAGGCAGTATCCAAAATCTACACCTGAGTTCCTTCTTTATATTTCTGATGGTGGAGCGTTAAGTTCCAACATTCCCACAAGAATGACTGCTAAAGTTAATCGAGATAATAAAGAAGTGGATCTGCTCATCTCCTCTGCTGTTGTATCAGACTCTGCACTATACTACTGTGCGCTGGTGNNNNNNACTGGAAG

Translation

PTGNYLHWYRQYPKSTPEFLLYISDGGALSSNIPTRMTAKVNRDNKEVDLLISSAVVSDSALYYCALV??TG

Fish H1 Vα TS32.34 - Jα TS32.34

AACCAGTCCTACAGGAAACTACCTGCACTGGTACAGGCAGTATCCAAAATCTACACCTGAGTTCCTTCTTTATATTTCTGATGGTGGAGCGTTAAGTTCCAACATTCCCACAAGAATGACTGCTAAAGTTAATCGAGATAATAAAGAAGTGGATCTGCTCATCTCCTCTGCTGTTGTATCAGACTCTGCACTATACTACTGTGCGCTGGTG

Translation

TSPTGNYLHWYRQYPKSTPEFLLYISDGGALSSNIPTRMTAKVNRDNKEVDLLISSAVVSDSALYYCALV

Fish H6 Vα TS32.34 - Jα TS32.34

CTGGTACAGGCNGTATCCNAAATCTACACCTGAGTTCCTTCTTTATATTTCTGATGGTGGAGCGTTAAGTTCCAACATTCCCACAAGAATGACTGCTAAAGTTAATCGAGATAATAAAGAAGTGGATCTGCTCATCTCCTCTGCTGTTGTATCAGACTCTGCACTATACTACTGTGCGCTGGTG

Translation

WYR?Y?KSTPEFLLYISDGGALSSNIPTRMTAKVNRDNKEVDLLISSAVVSDSALYYCALV
