# Supplementary material for: Rearranged T Cell Receptor Sequences in the Germline Genome of Channel Catfish Are Preferentially Expressed in Response to Infection
Source: Front Immunol. 2018 Sep 27;9:2117. doi: 10.3389/fimmu.2018.02117 (PMC6170632; doi:10.3389/fimmu.2018.02117)
Supplement: Supplementary file 3 [file Table_3.docx]

Supplemental Table 3. Channel catfish TCR Vβ to Jβ gene and Vα to Jα gene sequences amplified by PCR from DNA isolated from erythrocytes. Predicted CDR3 aa sequences are underlined. The fish from which the erythrocytes were isolated are shown.

**TCR Vβ**

Fish 51 Vβ1–Jβ13

GTGTACAGCTGCCAGTGGTCCAAATTAGAAGAAGGAGTTTACAAACGGTTCAAACTGAGCAGACAGGGCACGATTGCAGGAACTCTGACCATCTCTAATCTCCGCCAGTCAGACTCTGCTGTTTATTACTGTGCAGCCAGTCAATCAGGGAGGACTCAGGCCAGTCAAGCTTACTTCGGTGGAGGAACCAAGTTAACATATCT

Translation

VYSCQWSKLEEGVYKRFKLSRQGTIAGTLTISNLRQSDSAVYYCAASQSGRTQASQAYFGGGTKLTY

Fish 52 Vβ1–Jβ20

GGTATTGGCAAAACAGTTGGACAGTTAAGGCAATGATTGGATATACCACGACAGTTAAAAGTGATCCAAAATATGAGGAGGAATTTAACGATCGGTTCACACTGAGCAGACAGAGCACACTTGCAGGAACTCTGACCATCTCTAATCTCCGCCAGTCAGACTCTGCTGTTTATTACTGTGCAGCCAGTCAATCAGGGGAGGGNGGGTG

Translation

YWQNSWTVKAMIGYTTTVKSDPKYEEEFNDRFTLSRQSTLAGTLTISNLRQSDSAVYYCAASQSGEGG

Fish 7 Vβ2–Jβ24

ACACAGTTATGGCACTGATTGGATATACCTATACGGCTACGAGTAAGCCAGAGTACGAGGACGGATTTAATGTTAGGTACAAACAGAGCAGAAAGAGCATANCTGAAGGAAGTCTGACCATCTCTAAACTCCTCCAGTCAGACTCGGCTGTTTATTACTGTGCAGCCAGAATGCAGGGAGACACTCAGCCTGCATACTTTGG

Translation

TVMALIGYTYTATSKPEYEDGFNVRYKQSRKSI?EGSLTISKLLQSDSAVYYCAARMQGDTQPAYF

Fish 51 Vβ3–Jβ17

ACTGAGCCAAAGAACGAGGAAGATTTTAAAGATCGGTTCGAACAGAGCAGACAGATCATAATGGCAGGAAAACTTACCATCTCTAAAGTACTTCAGTCAGACTCTGCTGTTTATTACTGTGCAGCACAGGGTGGTGGTGCCAATCAAGCTTACTTTGGTGGAGGAACCAAGTTAACAGTTC

Translation

TEPKNEEDFKDRFEQSRQIIMAGKLTISKVLQSDSAVYYCAAQGGGANQAYFGGGTKLTV

Fish 52 Vβ3–Jβ17

ACTGAGCCAAAGAACGAGGAAGATTTTAAAGATCGGTTCGAACAGAGCAGACAGATCATAATGGCAGGAAAACTTACCATCTCTAAAGTACTTCAGTCAGACTCTGCTGTTTATTACTGTGCAGCACAGGGTGGTGGTGCCAATCAAGCTTACTTTGGTGGAGGAACCAAGTTAACAGTCC

Translation

TEPKNEEDFKDRFEQSRQIIMAGKLTISKVLQSDSAVYYCAAQGGGANQAYFGGGTKLTV

Fish 51 Vβ4–Jβ20

GACTTTGGAAAATCCGACCAAAAGAAATTTTCAGCTATTAAAACAGTTCCTGAGAACGGCTCATTCACAGTGAAAGACGTGGATTATAATGACAACGCTGTGTATTTCTGTGCCGTGAGAGAATTCTCTGGGGGCCGTGAAGCTTACTTTGGTGGAGGAACCAAGTTAACAG

Translation

DFGKSDQKKFSAIKTVPENGSFTVKDVDYNDNAVYFCAVREFSGGREAYFGGGTKLT

Fish 52 Vβ4–Jβ20

GACTTTGGAAAATCCGACCAAAAGAAATTTTCAGCTATTAAAACAGTTCCTGAGAACGGCTCATTCACAGTGAAAGACGTGGATTATAATGACAACGCTGTGTATTTCTGTGCCGTGAGAGAATTCTCTGGGGGCCGTGAAGCTTACTTTGGTGGAGGAACCAAGTTAACAGTTCT

Translation

DFGKSDQKKFSAIKTVPENGSFTVKDVDYNDNAVYFCAVREFSGGREAYFGGGTKLTV

Fish 7 Vβ5–Jβ27

GACACAGGATTTAAATACATGGGGTATCTGAACACTATTTTTCCAAAAGAAGAGGCGGAATTTGGAACGAAGATCAAGTTGAGTGGTGATGGGAGAAAGAGCGGCTCTATGACCATAAACAGTCTTTCCGTGAACGACAGTGCTGTATATTTCTGTGTAGCAGACAGGGGAGGGAGCCTTCAAGCTTACTTTGGTGAAGG

Translation

DTGFKYMGYLNTIFPKEEAEFGTKIKLSGDGRKSGSMTINSLSVNDSAVYFCVADRGGSLQAYFGE

Fish 7 Vβ5 like–Jβ20

CCAGGACACAGGATTTACACTCATGGGATACCTTTACACTCTTTCTTCAACGCTGGAGGAAGAATTTGAAACCAAAATCAAACTGAGTGGTGATGGGAGAAATAATGGTTCTTTGACCATAAAGAGTCTTTCAGTGAATGACAGTGCTGTGTAT

TTCTGTGCAGCTTATTATCACAGGGTTGGGGGCCGTGAAGCTTACTTTGGTGGAGGAACCAAGTTAACAG

Translation

QDTGFTLMGYLYTLSSTLEEEFETKIKLSGDGRNNGSLTIKSLSVNDSAVYFCAAYYHRVGGREAYFGGGTKLT

**TCR Vα**

Fish 51 Vα TS32.34 - Jα TS32.34

AACAACCAGTCCTACAGGAAACTACCTGCACTGGTACAGGCAGTATCCAAAATCTACACCTGAGTTCCTTCTTTATATTTCTGATGGTGGAGCGTTAAGTTCCAACATTCCCACAAGAATGACTGCTAAAGTTAATCGAGATAATAAAGAAGTGGATCTGCTCATCTCCTCTGCTGTTGTATCAGACTCTGCACTATACTACTGTGCGCTGGTGCCTACTACTGGAAGC

Translation

TTSPTGNYLHWYRQYPKSTPEFLLYISDGGALSSNIPTRMTAKVNRDNKEVDLLISSAVVSDSALYYCALVPTTGS

Fish 52 Vα TS32.34 - Jα TS32.34

AACAACCAGTCCTACAGGAAACTACCTGCACTGGTACAGGCAGTATCCAAAATCTACACCTGAGTTCCTTCTTTATATTTCTGATGGTGGAGCGTTAAGTTCCAACATTCCCACAAGAATGACTGCTAAAGTTAATCGAGATAATAAAGAAGTGGATCTGCTCATCTCCTCTGCTGTTGTATCAGACTCTGCACTATACTACTGTGCGCTGGTGCCTACTACTGGAAGC

Translation

TTSPTGNYLHWYRQYPKSTPEFLLYISDGGALSSNIPTRMTAKVNRDNKEVDLLISSAVVSDSALYYCALVPTTGS
