# Supplementary material for: Rearranged T Cell Receptor Sequences in the Germline Genome of Channel Catfish Are Preferentially Expressed in Response to Infection
Source: Front Immunol. 2018 Sep 27;9:2117. doi: 10.3389/fimmu.2018.02117 (PMC6170632; doi:10.3389/fimmu.2018.02117)
Supplement: Supplementary file 4 [file Table_4.docx]

Supplemental Table 4. Channel catfish TCR Vβ to Jβ gene sequences amplified by PCR from DNA isolated from oocytes with a frameshift or stop codon in the Dβ to Jβ junction sequence. The predicted CDR3 aa sequences preceding the frameshift or stop codon are underlined. The fish from which the oocytes were isolated are shown.

Fish 3 TB4–Jβ17 (Forward sequence only). Frameshift.

CTGCTGGAGCGTCGAGTGANCCAAGCTACGAGGNNGGATTTAAAGATCGGTTCAAACAGAGCAGACAGGGCACACTTAATGGAAGCCTGACCATCTCTAATCTTCGCCAGTCAGACTCTGCTGTTTATTACTGTGCAATCTTGGACAGGGGGTGGTGCCAATCAAGCTTACTTTGGTGGAGGAACC

Translation

AGASS?PSYE?GFKDRFKQSRQGTLNGSLTISNLRQSDSAVYYCAILDRGWCQSSLLWWRN

Fish H6 Vβ2–Jβ29 Stop codon.

TCCAGTGCAGTCACAATGACAATACCTTACAAACAATGTTATGGTACCTGCAAAACAGTAACACAGTTATGGCACTGATTGGATATACCTATACGGCTACGAGTAAGCCAGAGTACGAGGACGGATTTAATGTTAGGTACAAACAGAGCAGAAAGAGCATAACTGAAGGAAGTCTGACCATCTCTAAACTCCTCCAGTCAGACTCGGCTGTTTATTACTGTGCAGCCAAAGAGGGTTAACAACCAGCCTGCATACTTTGGCCAGGGGACCAAACTCACAGTTCT

Translation

QCSHNDNTLQTMLWYLQNSNTVMALIGYTYTATSKPEYEDGFNVRYKQSRKSITEGSLTISKLLQSDSAVYYCAAKEG*

Fish 9 Vβ2–Jβ24 Stop codon.

TCCAGTGCAGTCACAATGACAATACCTTACAAACAATGTTATGGTACCTGCAAAACAGTAACACAGTTATGGCACTGATTGGATATACCTATACGGCTACGAGTAAGCCAGAGTACGAGGACGGATTTAATGTTAGGTACAAACAGAGCAGAAAGAGCATAACTGAAGGAAGTCTGACCATCTCTAAACTCCTCCAGTCAGACTCGGCTGTTTATTACTGTGCAGCCAGACCGGACAGATAACACTCAGCCTGCATACTTCGGCCAGGGGACCAAACTCACAGTTCTTG

Translation

QCSHNDNTLQTMLWYLQNSNTVMALIGYTYTATSKPEYEDGFNVRYKQSRKSITEGSLTISKLLQSDSAVYYCAARPDR*

Fish 3 Vβ3–Jβ17 Frameshift.

AGGAAGATTTTAAAGATCGGTTCGAACAGAGCAGACAGAGCATAATGGCAGGAAAACTTACCATCTCTAAAGTACTTCAGTCAGACTCTGCTGTTTATTACTGTGCAGCACGGATAGGACAGGCGGCTCTGGTGGTGCCAATCAAGCTTACTTTGGTGGAGGAACC

Translation

EDFKDRFEQSRQSIMAGKLTISKVLQSDSAVYYCAARIGQAALVVPIKLTLVEE

Fish 9 Vβ3–Jβ17 Frameshift.

GGAAGATTTTAAAGATCGGTTCGAACAGAGCAGACAGAGCATAATGGCAGGAAAACTTACCATCTCTAAAGTACTTCAGTCAGACTCTGCTGTTTATTACTGTGCAGCACGGATAGGACAGGCGGCTCTGGTGGTGCCAATCAAGCTTACTTTGGTGGAGGAACC

Translation

EDFKDRFEQSRQSIMAGKLTISKVLQSDSAVYYCAARIGQAALVVPIKLTLVEE
