# Supplementary material for: Rearranged T Cell Receptor Sequences in the Germline Genome of Channel Catfish Are Preferentially Expressed in Response to Infection
Source: Front Immunol. 2018 Sep 27;9:2117. doi: 10.3389/fimmu.2018.02117 (PMC6170632; doi:10.3389/fimmu.2018.02117)
Supplement: Supplementary file 5 [file Table_5.DOCX]

Supplemental Table 5. Rearranged TCR Vβ sequence in the germline with leader, intron and Vβ gene sequences for clonotypes Vβ2 CAAHRGANPAYF and Vβ4 CAVREFSGGREAYF. Sequences were amplified by PCR from DNA isolated from oocytes. The junctions of the leader, intron and Vβ sequences are underlined. The fish from which the oocytes were isolated are indicated.

1. **Vβ2 CAAHRGANPAYF**

Fish B11

TTTTTTTTTCTTTAAATTGGATTTTATGGGCCCCGGGATTTGATTCGGAATTAAAAAACCTGATTGGGGGTTGACAACTATTTCCTTTTTTCCTCAGGGAGGATAAACTGTGTCAAATTTCAAACGATCTCATCTCTCCTAGTCAATGAAACAGAAGAAGTAACAATCCAGTGCAGTCACAATGACAATACCTTACAAACAATGTTATGGTACCTGCAAAACAGTAACACAGTTATGGCACTGATTGGATATACCTATACGGCTACGAGTAAGCCAGAGTACGAGGACGGATTTAATGTTAGGTACAAACAGAGCAGAAAGAGCATAACTGAAGGAAGTCTGACCATCTCT

2. **Vβ4 CAVREFSGGREAYF**

Fish A22

CAGTCACTTTACTGGATTCAAGGAGAATACAAAACACACTGAATGCATCTGTAATAATAAGGAGTTACATTATTATCATAGTAATACTACAAATATTATACATGCCTAGGAATCAGAATATAAAATGAACAGCTAGCTTACATATCTTAAATCAACCCATTACTTACTGTTTATTCACAGGAGTTGCAGGGGCAAATGATGTCTTGCAGCCCGAAATACTCTGGGCTCAATTCGGCCAATCAGTCACAATTAACTGCTCACACACCAAGGGTTCAGCTTACAGAGAAATGTACTGGTTTCGTCAGTATCAAGGAGAGAGTATGGAGCTCATCGTGTACACTACCAGCTTTGGCACTCCAGACTTTGGAAAATCCGACCAAAAGAAATTTTCAGCTATTAAAACAGTTCCTGAGAACGGCTCATTCACAGTGAAAGACGTGGATTATAATGACAACGTGTGTATTTCTGTGCCGTGAGAGAATTCTCTG

3. **Vβ4 CAVREFSGGREAYF**

Fish B11-2

CAGTCACCTTACTGGATTCAAGGATACTACAAAACACACTGNATGCGTTTGTTATAATAAGGAGTTACATTATTATCATAGTAATGNTACAAATATTATACATGCNTAGGAATCAAAATATAAAATGAACAGCTAGCTTACATATNTTAAATCAACCCATTACTTACTGTTTATTCACAGGAGTTGCAGGGGCAAATGATGTCTTGCAGCCCGATATACTCTGGGCTCAATTGGGCCAATCAGTCACAATTAACTGCTCACACACCAAGGGTTCAGTTTACAGAGAAATGTACTGGTTTCGTCAGTATCAAGGAGAGAGTATGGAGCTCATCGTGTACACTACCAGCTTTGGCACTCAAGACTTTGGAAAATCCGACCAAAAGAAATTCTCAGCTATTAAAACAGTTCCTGAGAAGCGGCTCATNCACAGTGAAAGA
